# Use of Ether

**Published:** 1890-08-30

**Authors:** 


### THERAPEUTICS.

### USE OF ETHER.

Sir James Sawyer, Professor of Medicine at the Queen's College, Birmingham, has some remarks in a recent number of the *Lancet* on ether as a menstruum in medication of the skin. Authorities have been divided as to how far the local and remote effects of remedies applied to the human body in morbid states can be produced through the cutaneous surface. There are three separate obstacles to the absorption of a medicine through the skin, viz., the epidermis, the sebaceous secretion of the skin, and the relative insensibility of the drug employed in any particular case. Ether, Sir James Sawyer says, presents advantages over other bases and menstrua for remedies applied to the skin. "It has a great endosmotic capacity, it probably possesses in a high degree what has been called diffusion-power, it is a solvent of high potency for many active drugs, or more precisely for the active principles of many such drugs, and it is also a ready solvent for the sebaceous secretion of the skin." Ethereal tincture of capsicum is a very active rubefacient, and an efficient remedy in several painful maladies. In addition to this, Sir J. Sawyer mentions belladonna, iodine, and menthol as suitable for employment in the form of ethereal tinctures. Ethereal tincture of belladonna should be made from belladonna root, with camphor of the same strength as the belladonna liniment of the pharmacopœia, using the officinal pure ether in its preparation instead of rectified spirits of wine. This tincture is useful as a paint to the skin in cardiac and other cases in which belladonna plaster or liniment would be employed. Ethereal tincture of iodine is made of the same strength as the officinal tincture of iodine. Ethereal tincture of menthol is one drachm of menthol in a fluid ounce of pure ether. This is especially useful for superficial neuralgic pains. It should be lightly painted over the painful part with a glass brush. The quick evaporation of the ether gives a grateful sense of coolness, which supplements the analgesic action of the menthol.

